# Supplementary material for: Synthesis of Tricyclic Pterolobirin H Analogue: Evaluation of Anticancer and Anti-Inflammatory Activities and Molecular Docking Investigations
Source: Molecules. 2023 Aug 23;28(17):6208. doi: 10.3390/molecules28176208 (PMC10489156; doi:10.3390/molecules28176208)
Supplement: Supplementary file 1 [file molecules-28-06208-s001.zip › molecules-2539462-supplementary.pdf]

SUPPORTING INFORMATION

Synthesis of a Tricyclic Pterolobirin H Analogue: Evaluation of Anticancer and Anti-inflammatory Activities and Molecular Docking Investigations

Houda Zentar [1,2], Fatin Jannus [2], Marta Medina-O'Donnell [2], Az-eddine El Mansouri [3], Antonio Fernández [1], José Justicia [1], Enrique Alvarez-Manzaneda [1], Fernando J. Reyes-Zurita [2,*] and Rachid Chahboun [1,*]

[1] Departamento de Química Orgánica, Facultad de Ciencias, Instituto de Biotecnología, Universidad de Granada, 18071 Granada, Spain; zentarhouda@correo.ugr.es (H.Z.); ajfvargas@ugr.es (A.F.); jjusti@ugr.es (J.J.); eamr@ugr.es (E.A.-M.)
[2] Departamento de Bioquímica y Biología Molecular I, Facultad de Ciencias, Universidad de Granada, 18071 Granada, Spain; fatin@correo.ugr.es (F.J.); mmodonnell@ugr.e (M.M.-O.)
[3] Chemistry Department, University of the Free State, P.O. Box 339, Bloemfontein 9300, South Africa; elmansouri.a@ufs.ac.za
* Correspondence: ferjes@ugr.es (F.J.R.-Z.); rachid@ugr.es (R.C.); Fax: +34-958-248437 (R.C.)

Table of contents

Figure S1. ....S2

Table S1. ....S3

Figure S2. ....S4

Table S2. ....S5

Figure S3. ....S6

Figure S4. ....S7

^{1}H and ^{13}C NMR spectra of compounds. S8-S19

Infrared spectra of Compounds 12, 13, 14, 15, and 16...............................S20-S25

HRMS spectra of compounds 12,13, 14, 15, and 16.................................S26-S30

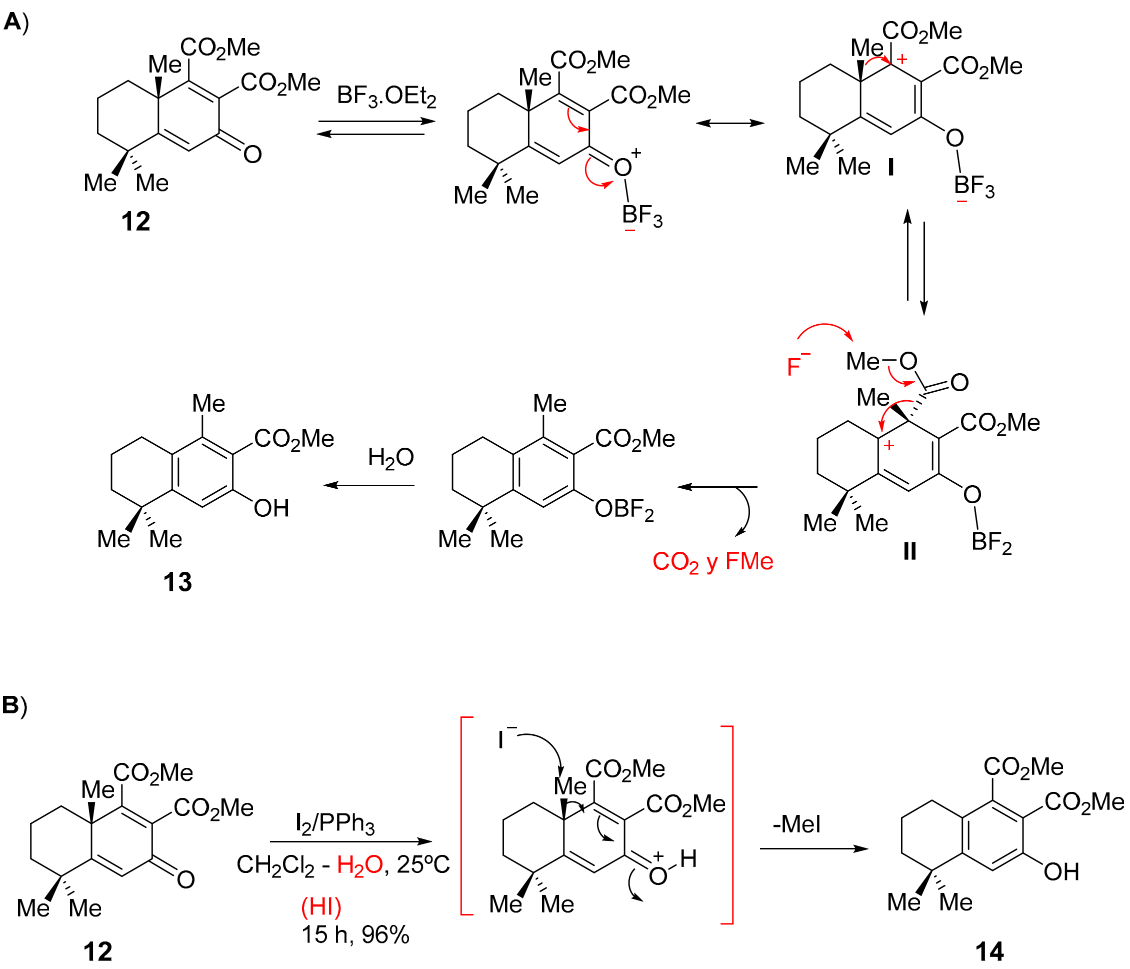

Figure S1. Proposed Mechanisms for the obtention of phenols **13** (A) and **14** (B) from dienone **12**.

Compound	Simplified analogue
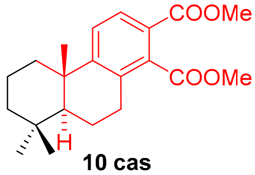<br>**10 cas**	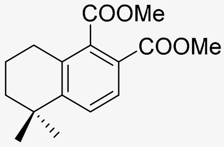<br>**10**
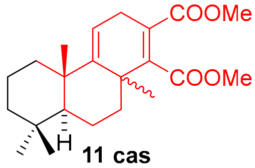<br>**11 cas**	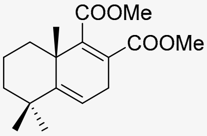<br>**11**
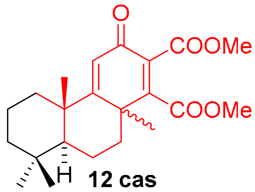<br>**12 cas**	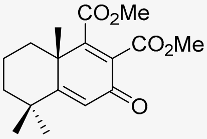<br>**12**
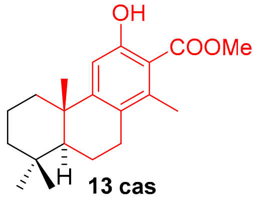<br>**13 cas**	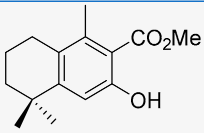<br>**13**
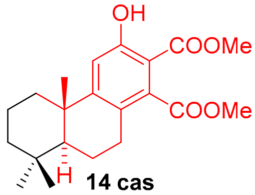<br>**14 cas**	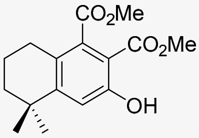<br>**14**
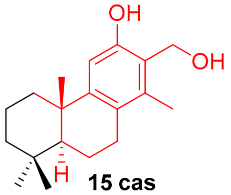<br>**15 cas**	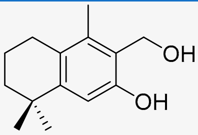<br>**15**
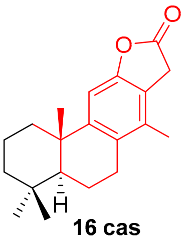<br>**16 cas**<br>Pterolobirin H (**3**)	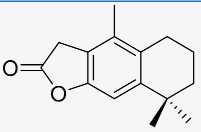<br>Simplified analogue of pterolobirin H -**16**

Table S1. Pterolobirin H (**3**), its intermediates from our previous report [1] and the simplified analogues.

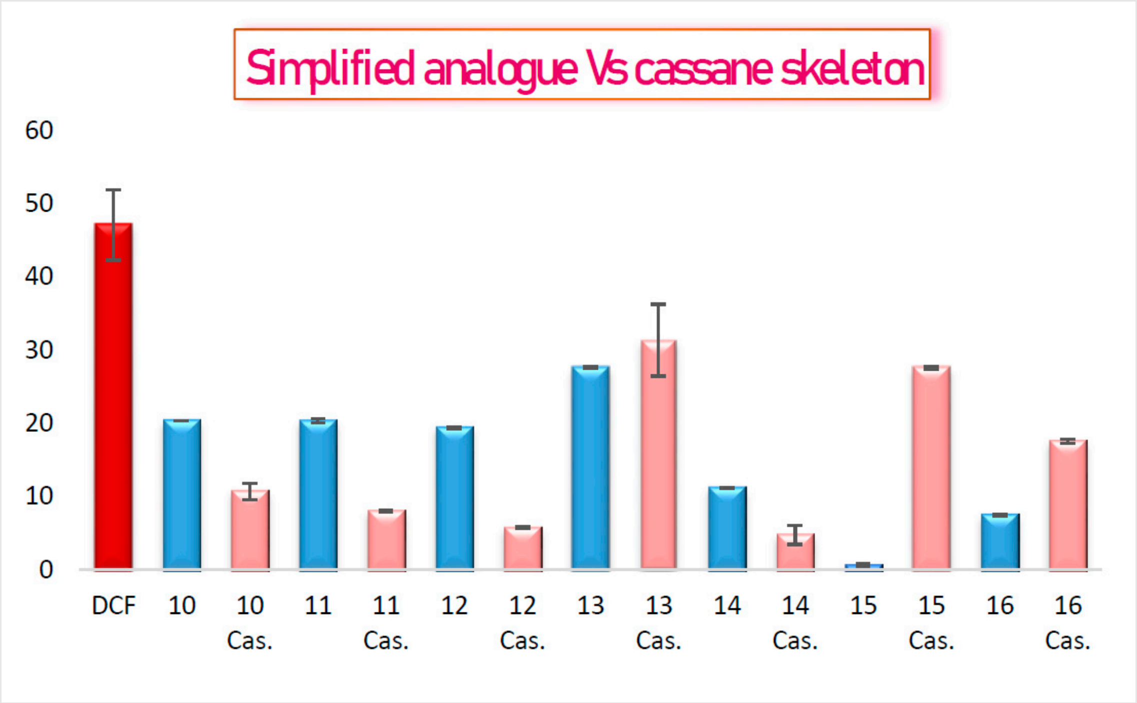

Figure S2. NO release-inhibitory effects $IC_{50\ NO}$ (µg/mL) concentrations for DCF, simplified analogues (**10-16**), and correspondent cassane diterpenoids (**10 cas -16 cas/ pterolobirin H (3)**). The data represent the mean ± SD of at least two independent experiments performed in triplicate.

**Simplified analogue**	**IC_{50NO} (µg/mL)**	**Cassane skeleton**	**IC_{50NO} (µg/mL)**
**10**	20.37 ± 0.01	**10 Cas.**	10.71 ± 1.14
**11**	20.34 ± 0.30	**11 Cas.**	7.98 ± 0.10
12	19.44 ± 0.12	**12 Cas.**	5.71 ± 0.14
**13**	27.60 ± 0.13	**13 Cas.**	31.37 ± 4.90
**14**	11.18 ± 0.09	**14 Cas.**	4.74 ± 1.27
**15**	**0.62 ± 8.21**	**15 Cas.**	**27.57 ± 0.20**
**16**	**7.49 ± 0.15**	**16 Cas. (pterolobirin H (3))**	**17.57 ± 0.31**

Table S2. The $IC_{50\ NO}$ values of cassanes **10** cas–**16** cas, and their simplified analogues **10–16**.

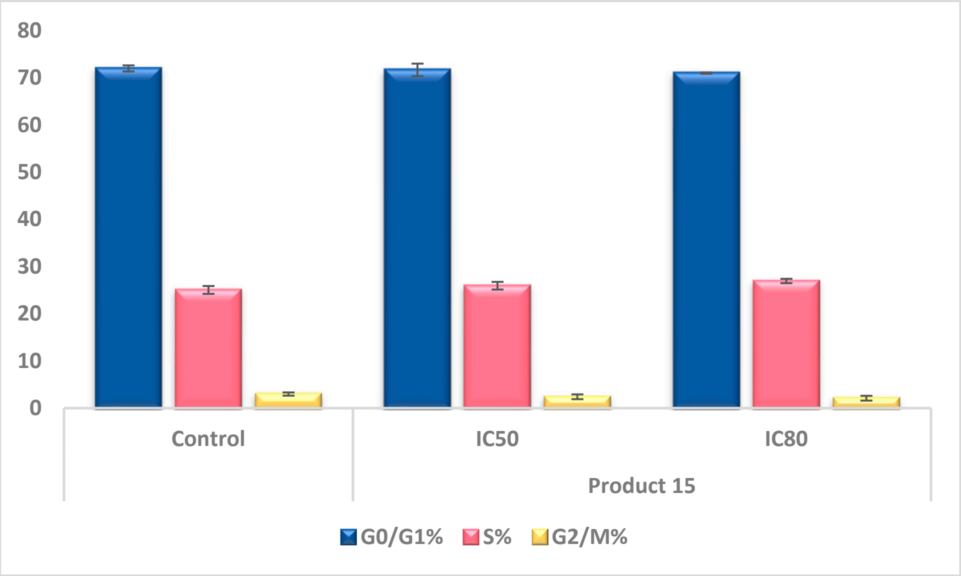

Figure S3. Top: Percentages of B16-F10 skin-melanoma cells in each cell-cycle phase, after 72 h treatment with compound **15**, at IC_{50} and IC_{80} concentrations. G0/G1 phase (Blue), S phase (pink), and G2/M phase (peach). Control (untreated cells); samples (cells treated with compounds **15**). Values represent means ± S. D. of at least three independent experiments performed in duplicate.

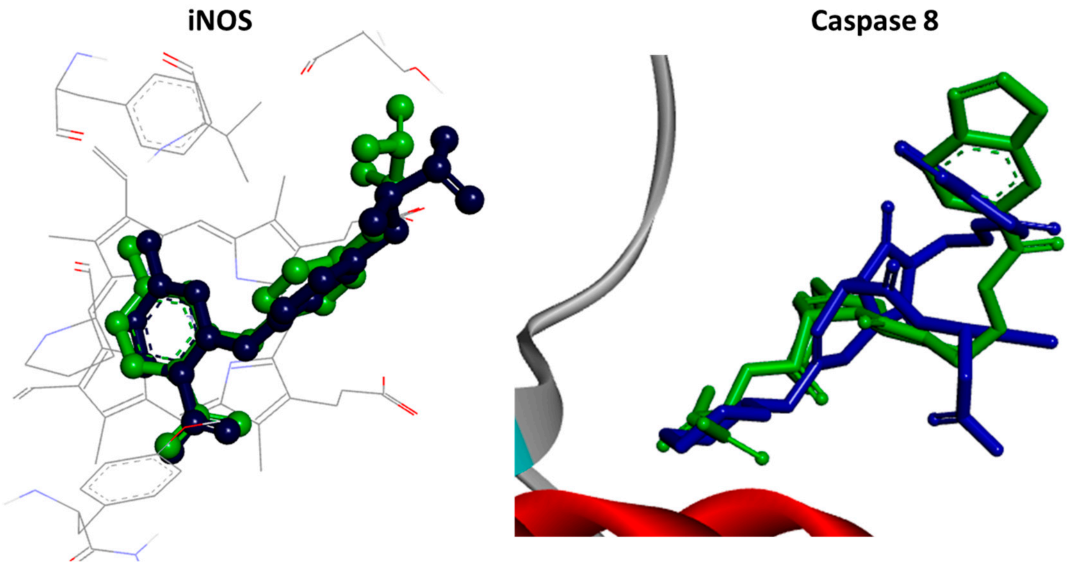

Figure S4. Poses of cognate YWO ligand on 4UX6 crystal structure (blue) and redocked (green).

.

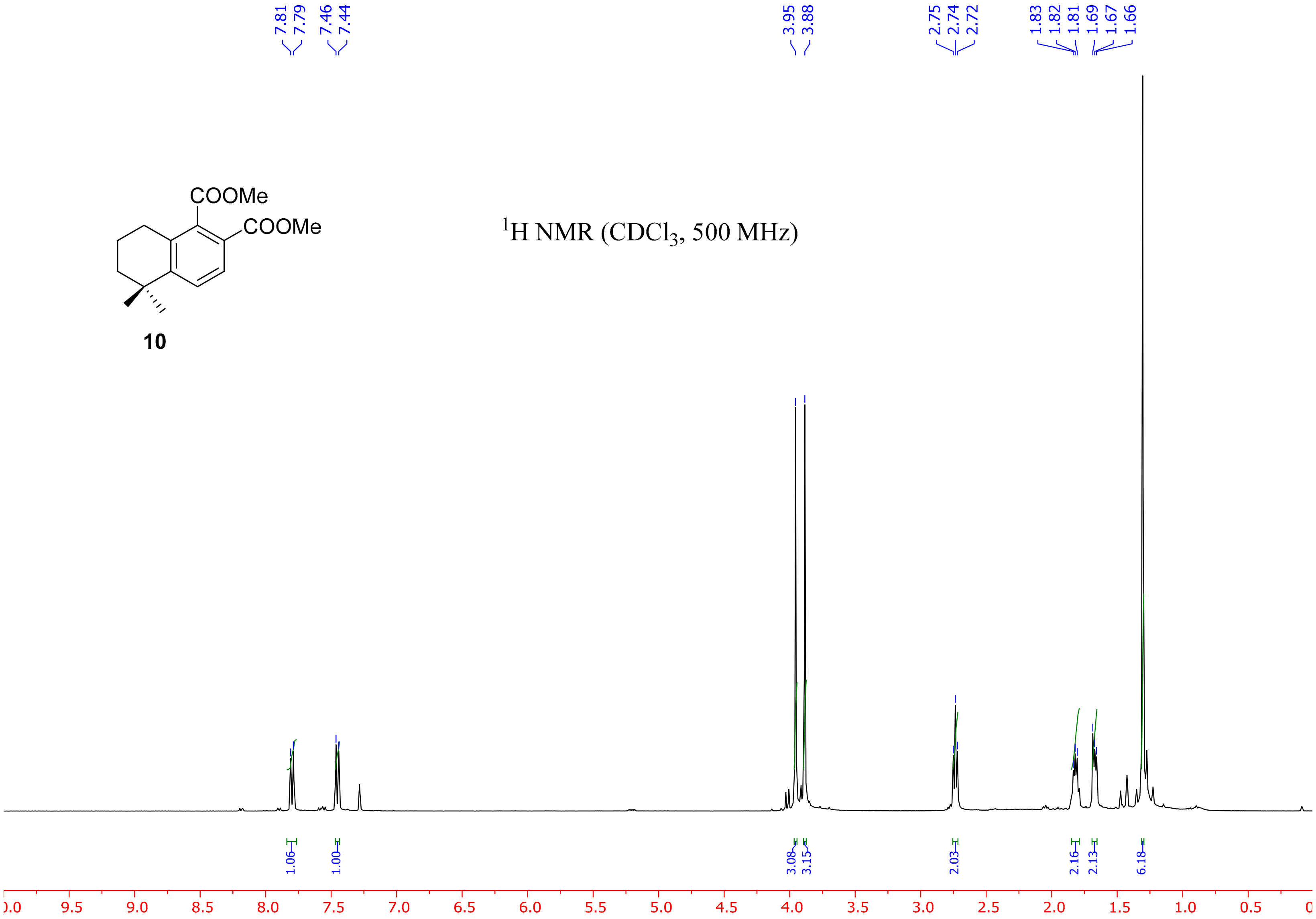
COOMe
COOMe
10
^{1}H NMR ($CDCl_3$, 500 MHz)
7.81
7.79
7.46
7.44
3.95
3.88
2.75
2.74
2.72
1.83
1.82
1.81
1.69
1.67
1.66
1.06
1.00
3.08
3.15
2.03
2.16
2.13
6.18
0.0
9.5
9.0
8.5
8.0
7.5
7.0
6.5
6.0
5.5
5.0
4.5
4.0
3.5
3.0
2.5
2.0
1.5
1.0
0.5
0

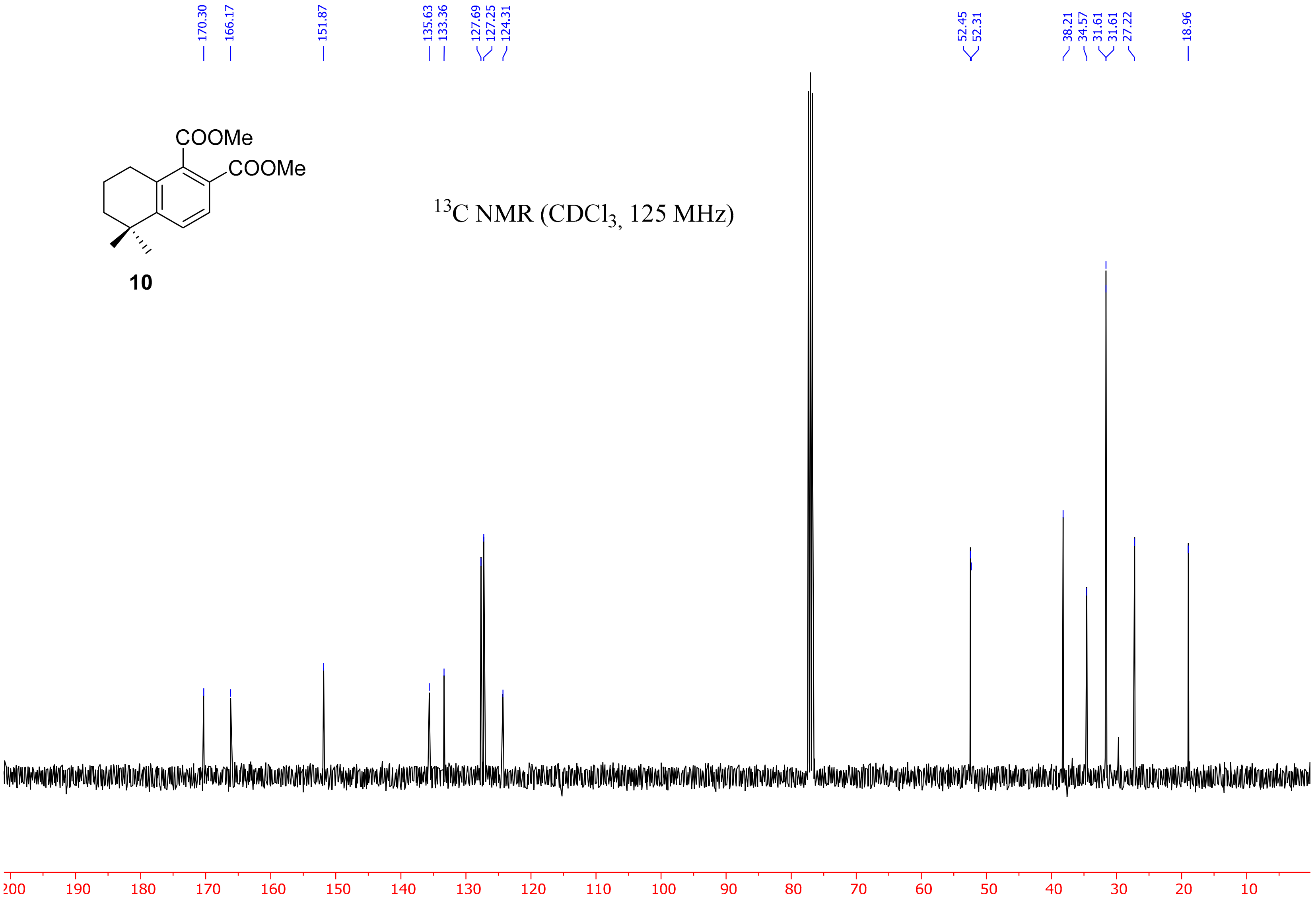

10

^{13}C NMR ($CDCl_3$, 125 MHz)

^{1}H NMR ($CDCl_3$, 500 MHz)

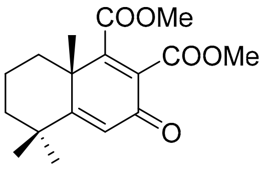

12

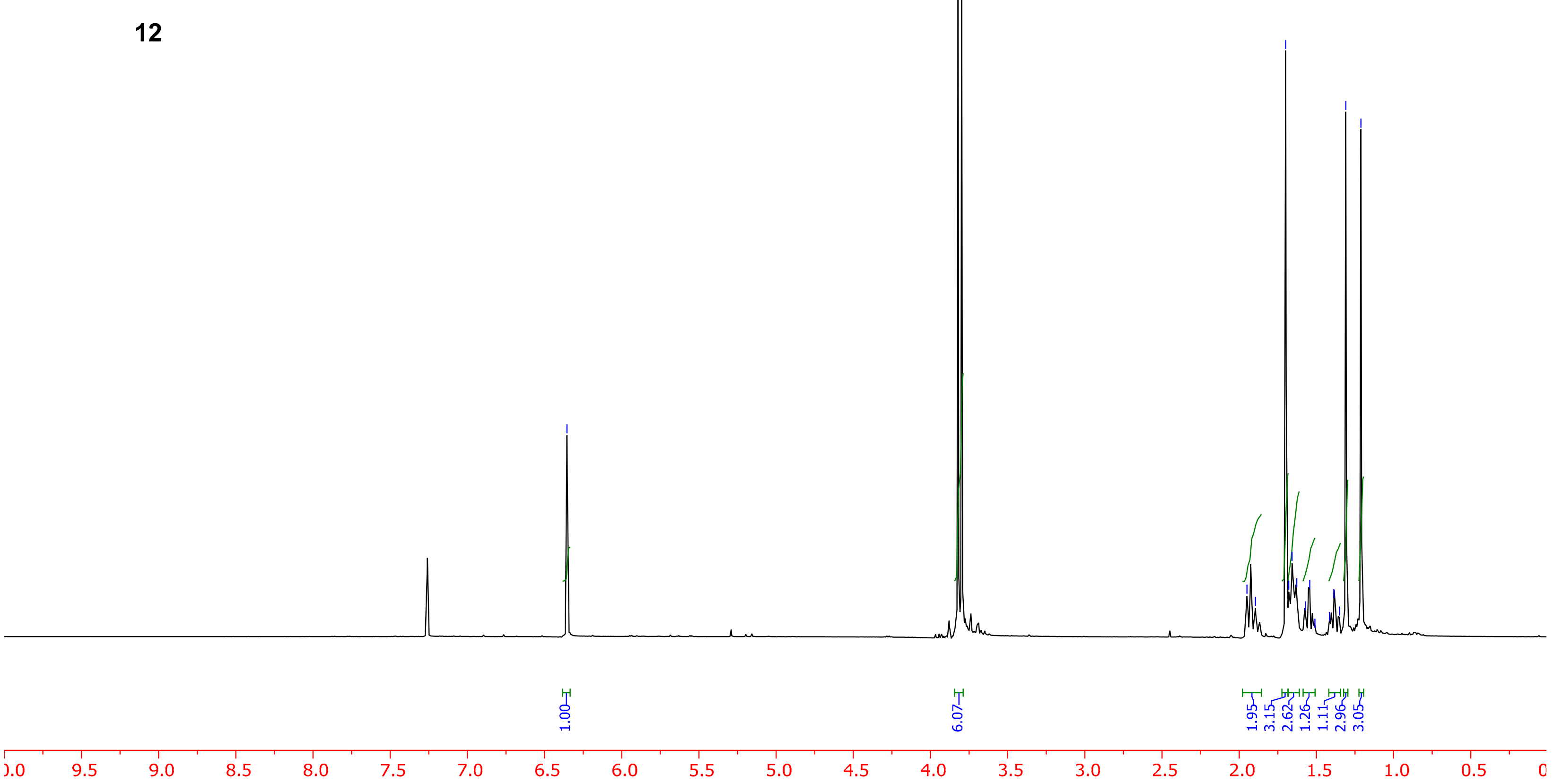

^{13}C NMR ($CDCl_3$, 125 MHz)

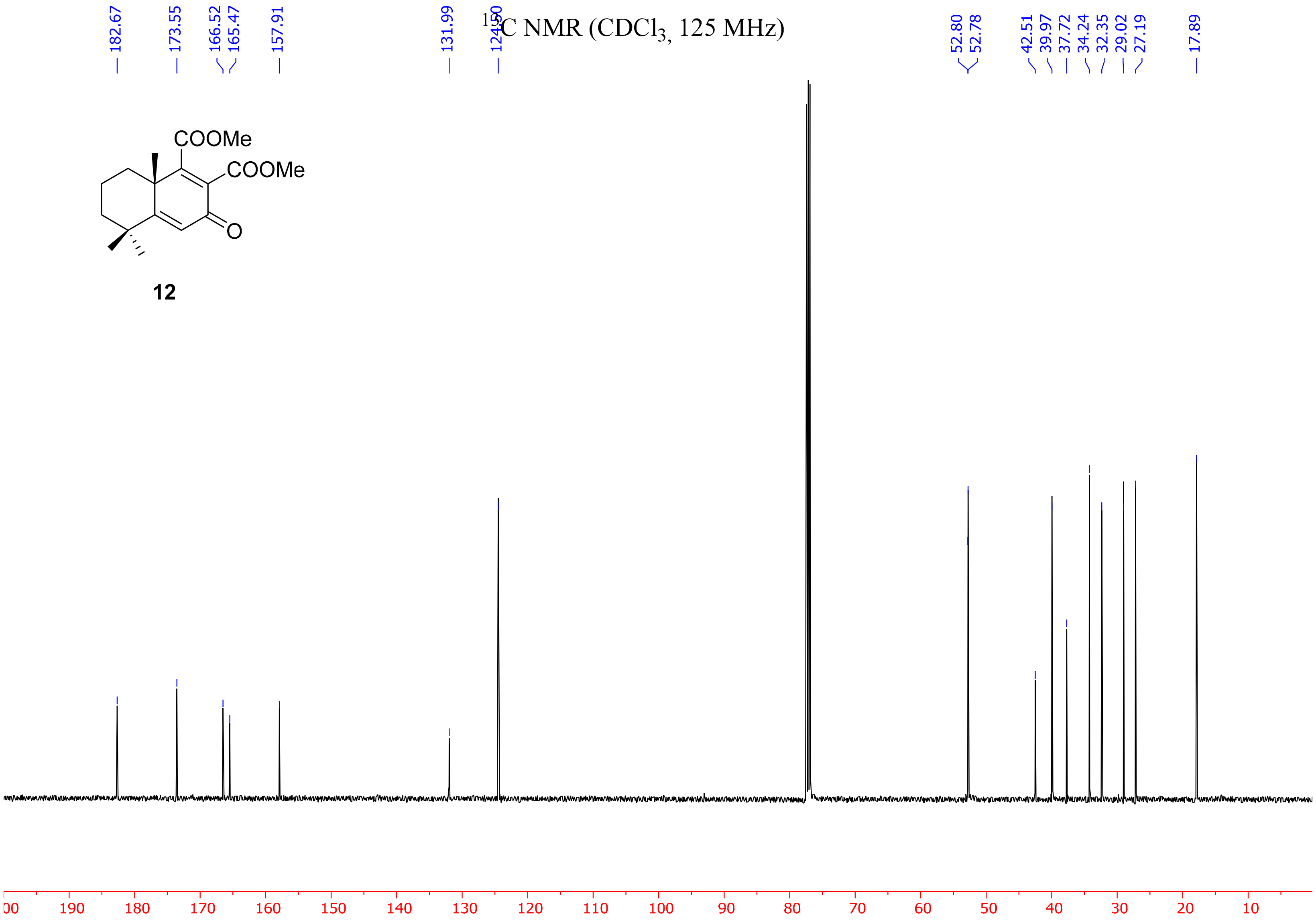

^{1}H NMR ($CDCl_3$, 500 MHz)

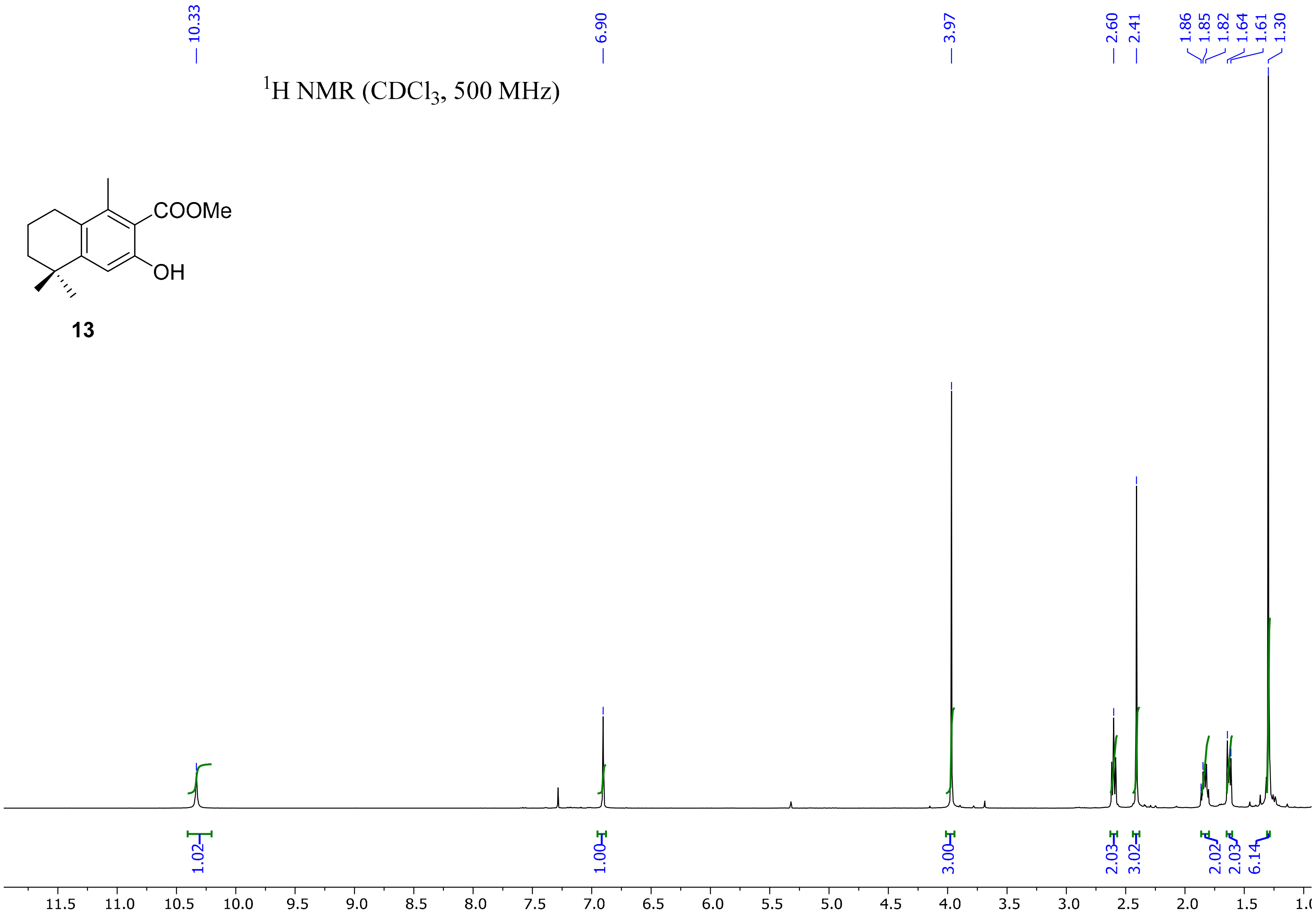

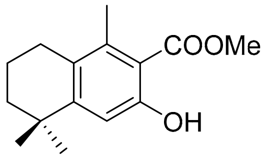

13

^{13}C NMR ($CDCl_3$, 125 MHz)

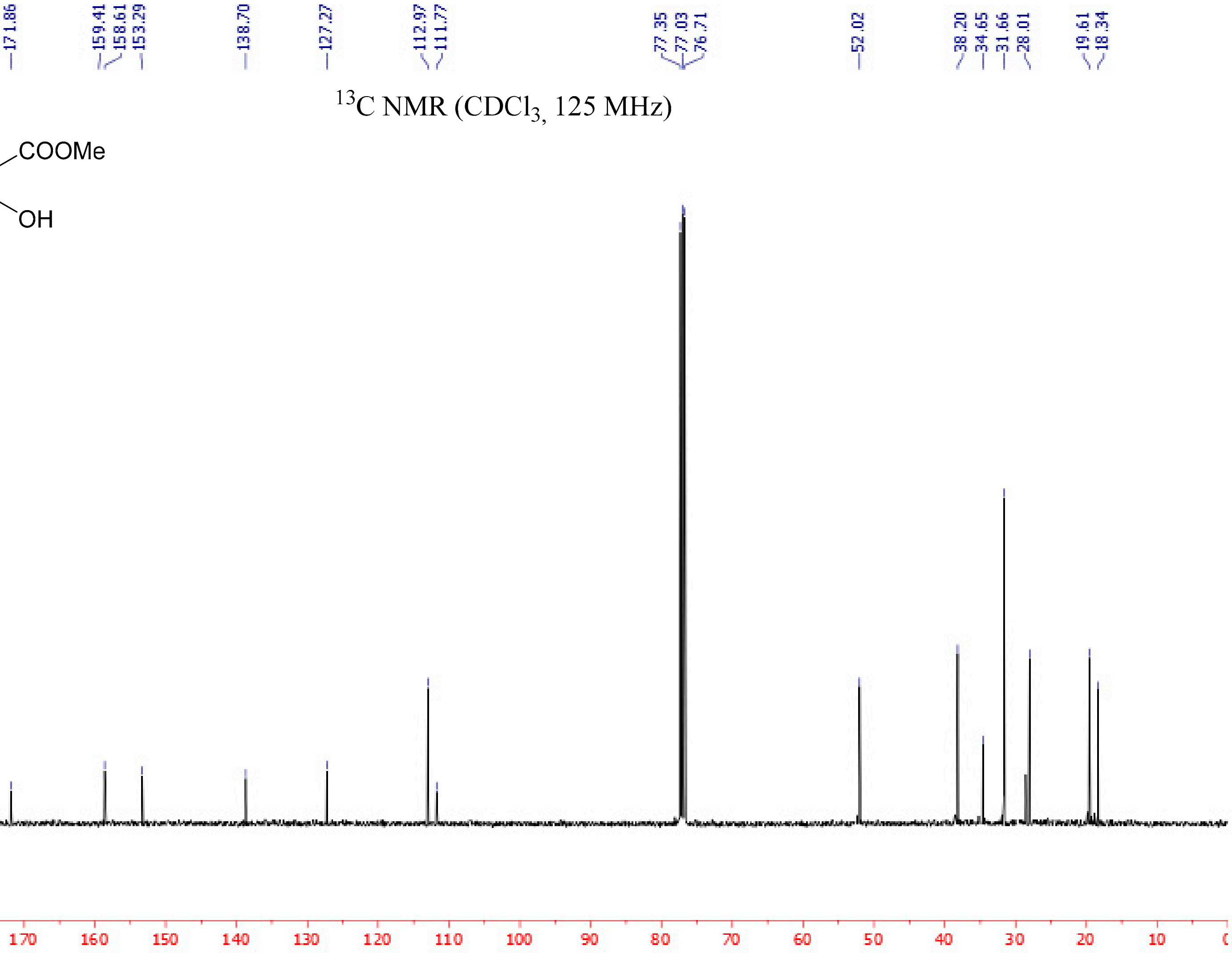

^{1}H NMR ($CDCl_3$, 500 MHz)

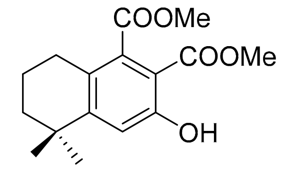

14

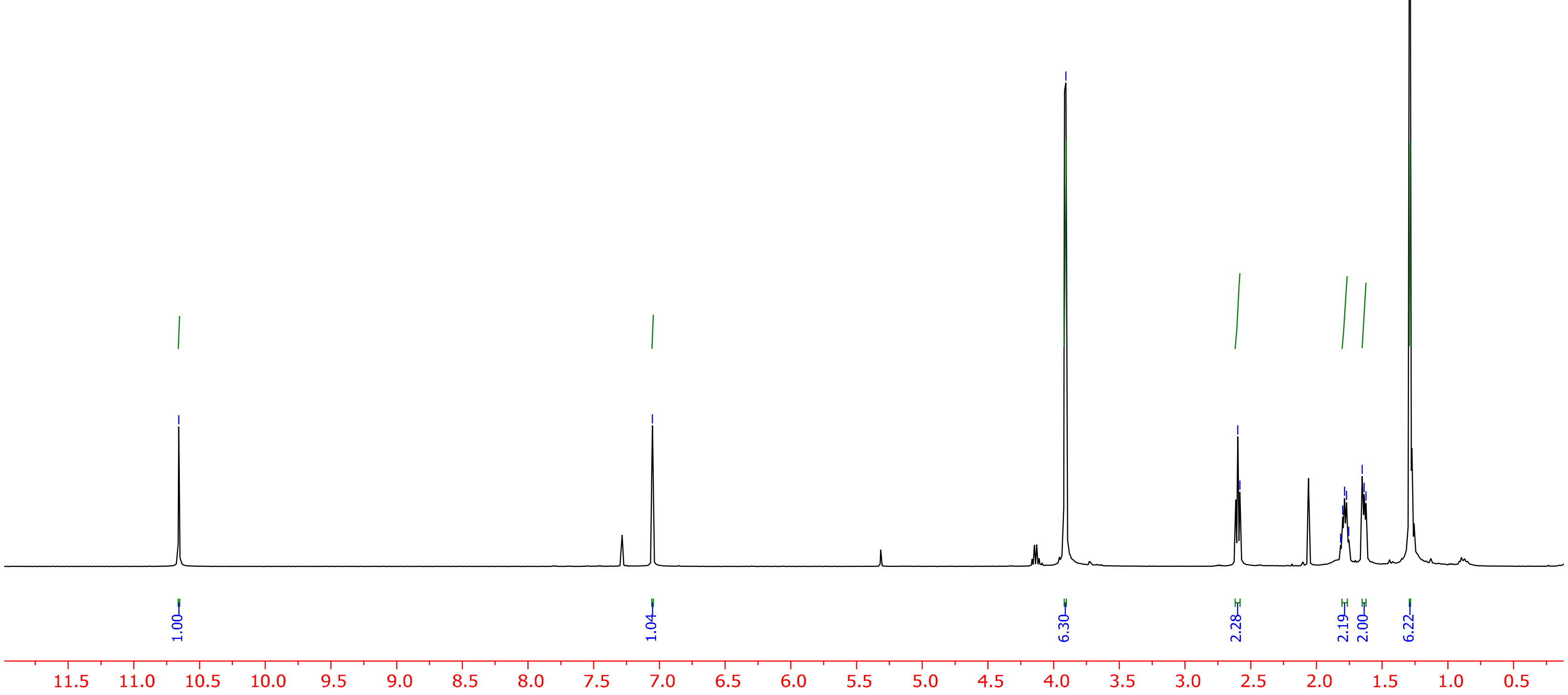

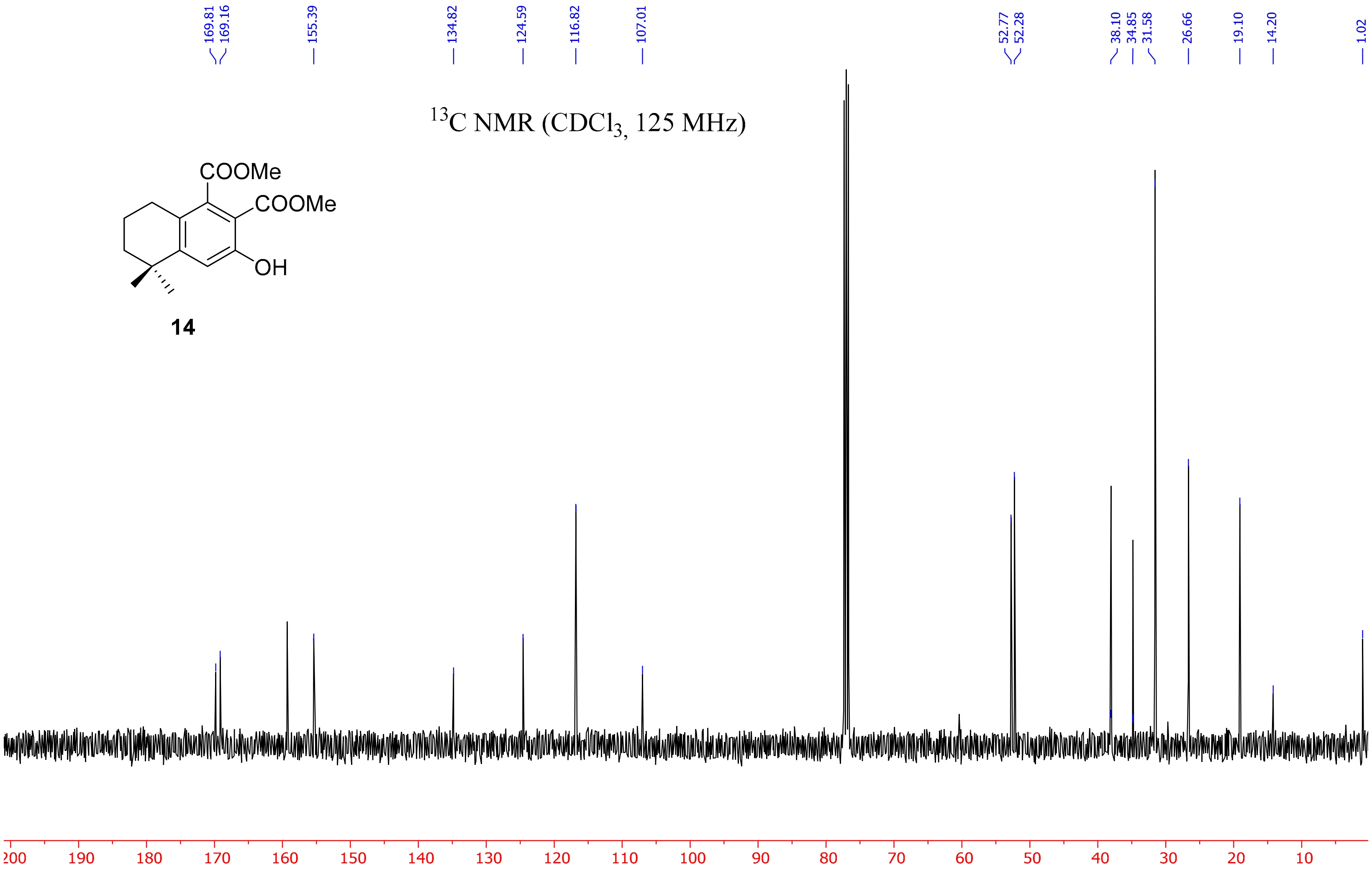
COOMe
COOMe
OH
14
^{13}C NMR (CDCl$_3$, 125 MHz)
169.81
169.16
155.39
134.82
124.59
116.82
107.01
52.77
52.28
38.10
34.85
31.58
26.66
19.10
14.20
1.02
200
190
180
170
160
150
140
130
120
110
100
90
80
70
60
50
40
30
20
10

^{1}H NMR (CDCl$_3$, 500 MHz)

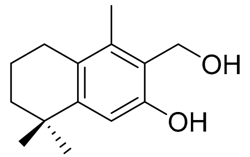

15

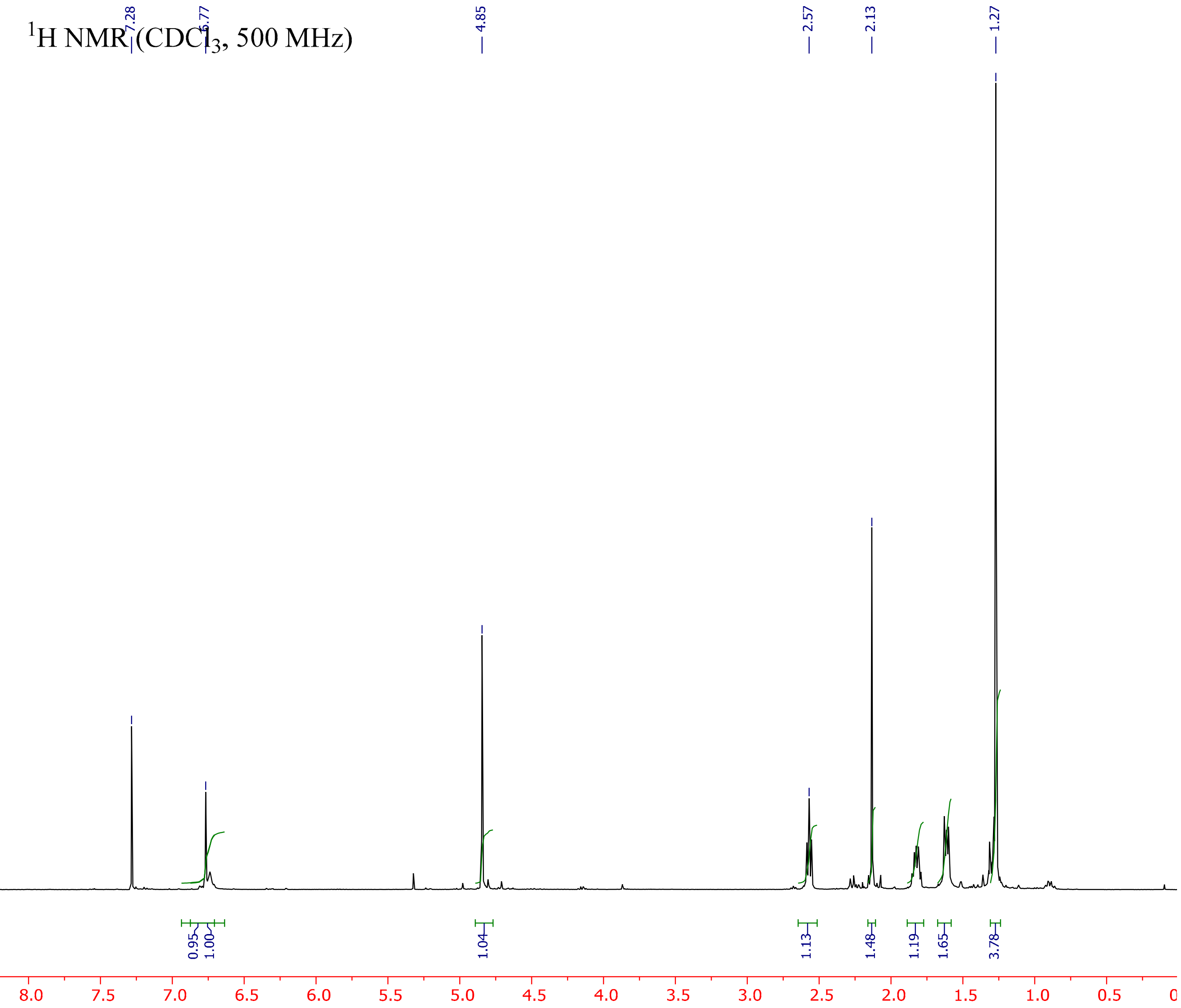

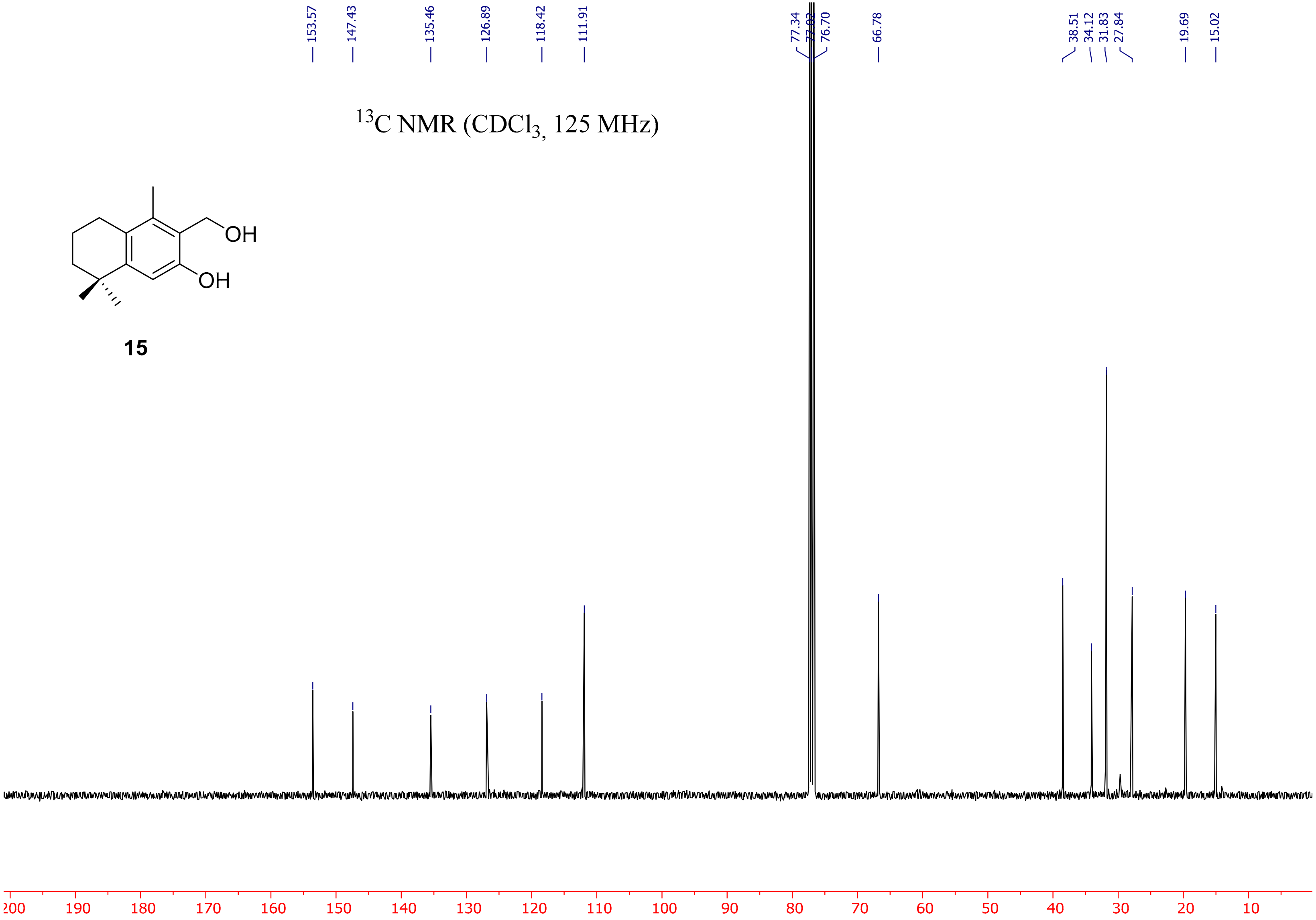
153.57
147.43
135.46
126.89
118.42
111.91
77.34
76.70
66.78
38.51
34.12
31.83
27.84
19.69
15.02
^{13}C NMR ($CDCl_3$, 125 MHz)
OH
OH
15
200 190 180 170 160 150 140 130 120 110 100 90 80 70 60 50 40 30 20 10

^{1}H NMR ($CDCl_3$, 500 MHz)

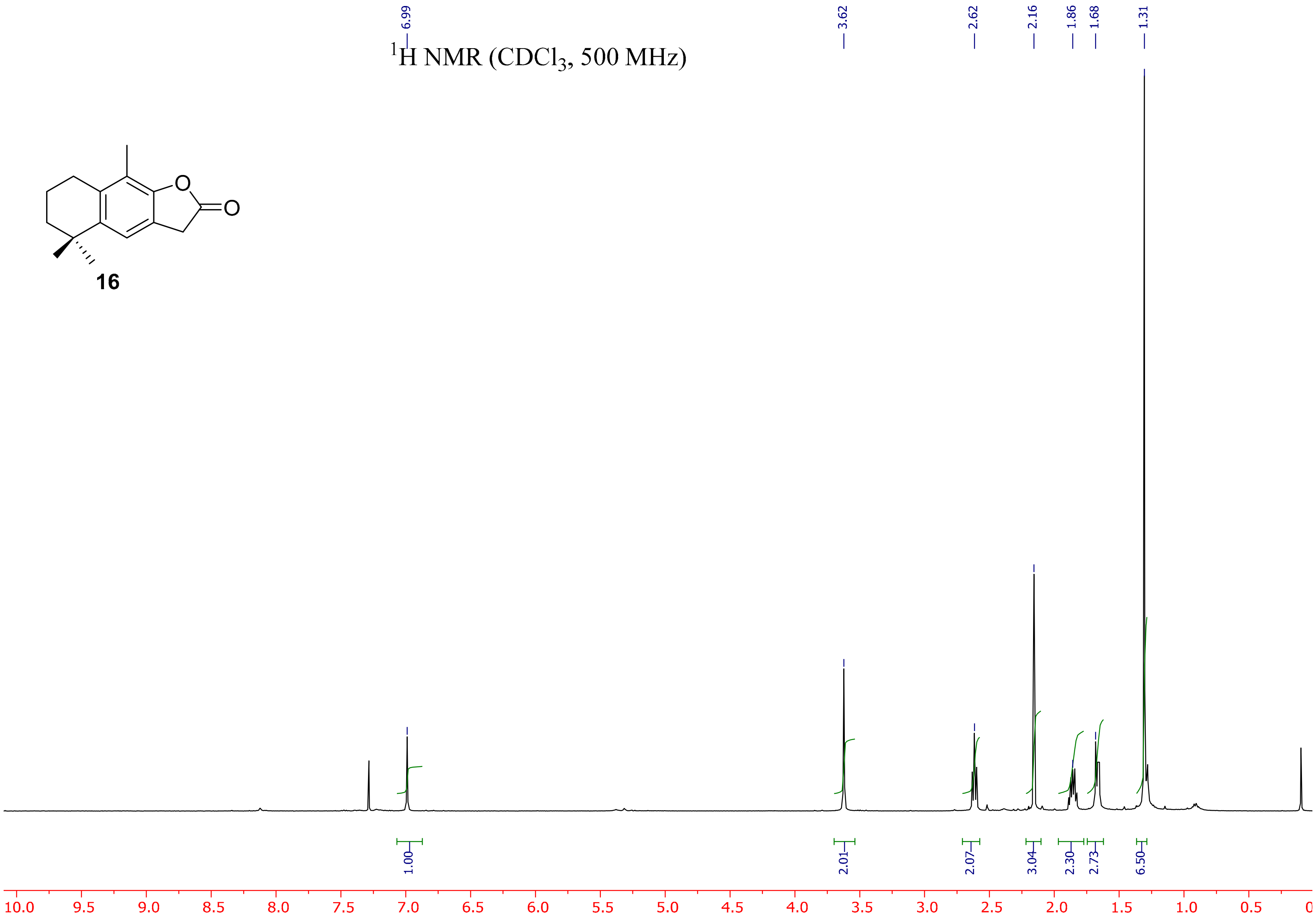

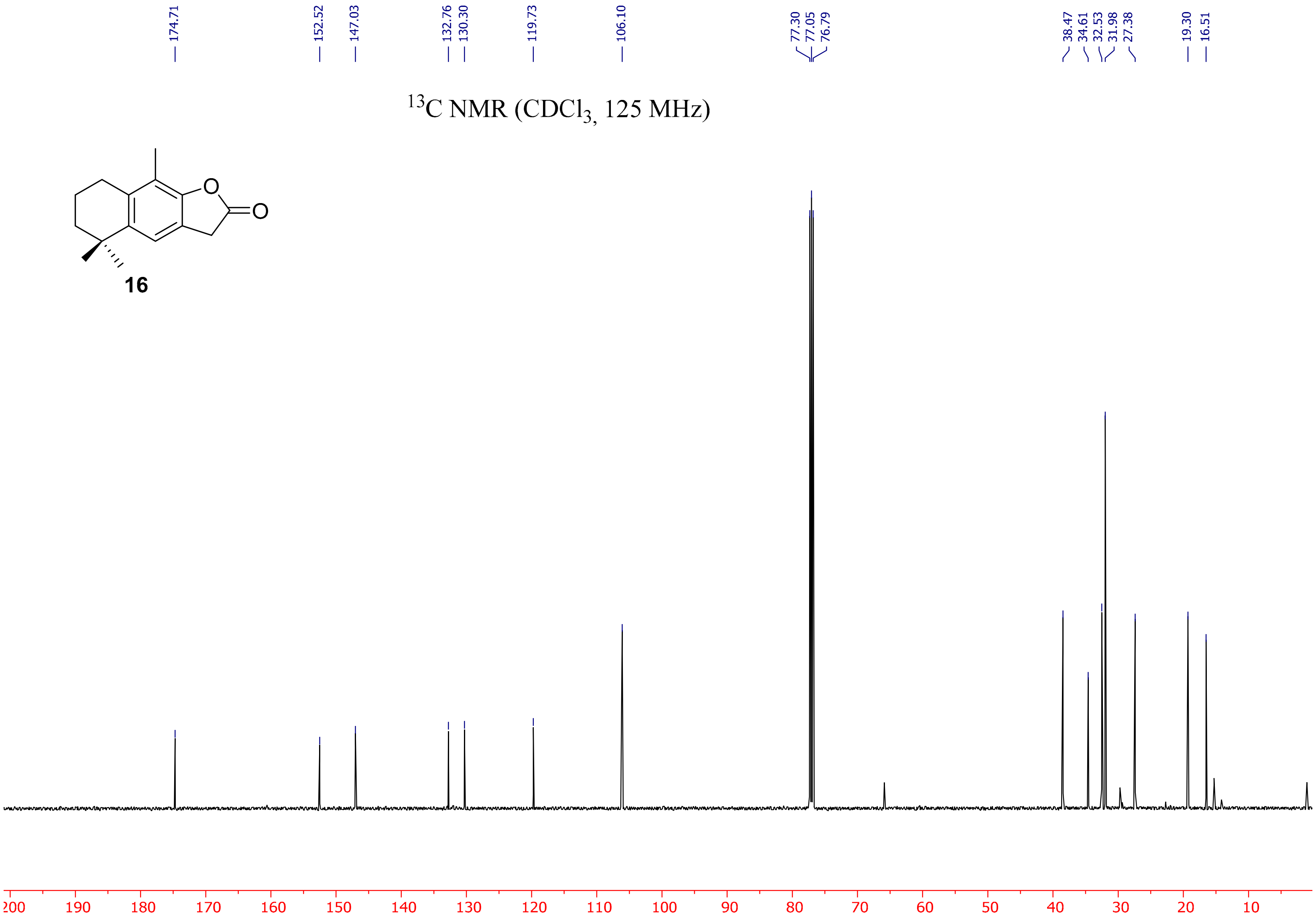
174.71
152.52
147.03
132.76
130.30
119.73
106.10
77.30
77.05
76.79
38.47
34.61
32.53
31.98
27.38
19.30
16.51
^{13}C NMR ($CDCl_3$, 125 MHz)
16
200 190 180 170 160 150 140 130 120 110 100 90 80 70 60 50 40 30 20 10

Infrared spectra of Compounds 10, 12, 13, 14, 15, and 16

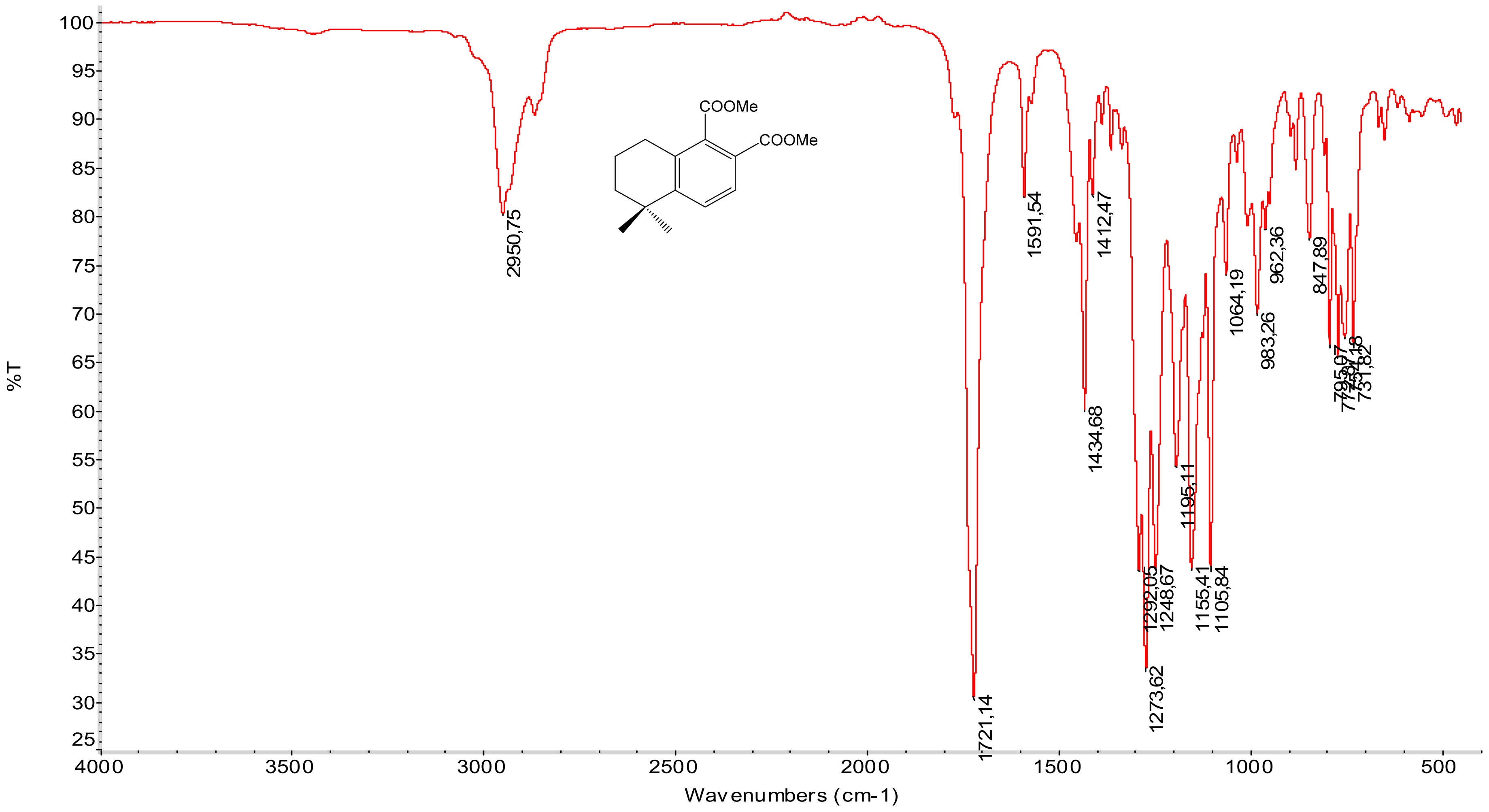

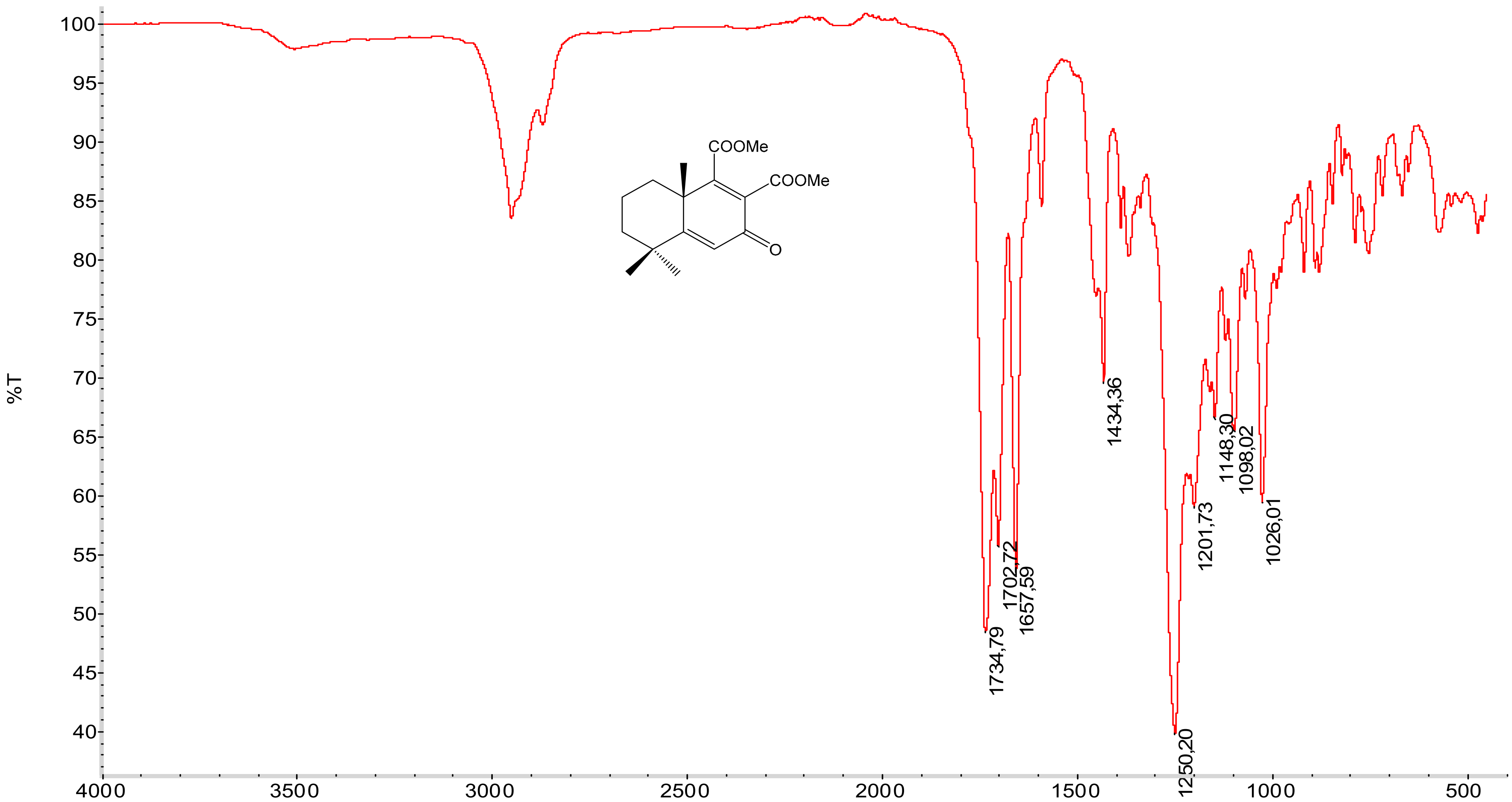

100
95
90
85
80
75
70
65
60
55
50
45
40
%T
4000
3500
3000
2500
2000
1500
1000
500
Wavenumbers (cm-1)
COOMe
COOMe
O
1734,79
1702,72
1657,59
1434,36
1250,20
1201,73
1148,30
1098,02
1026,01

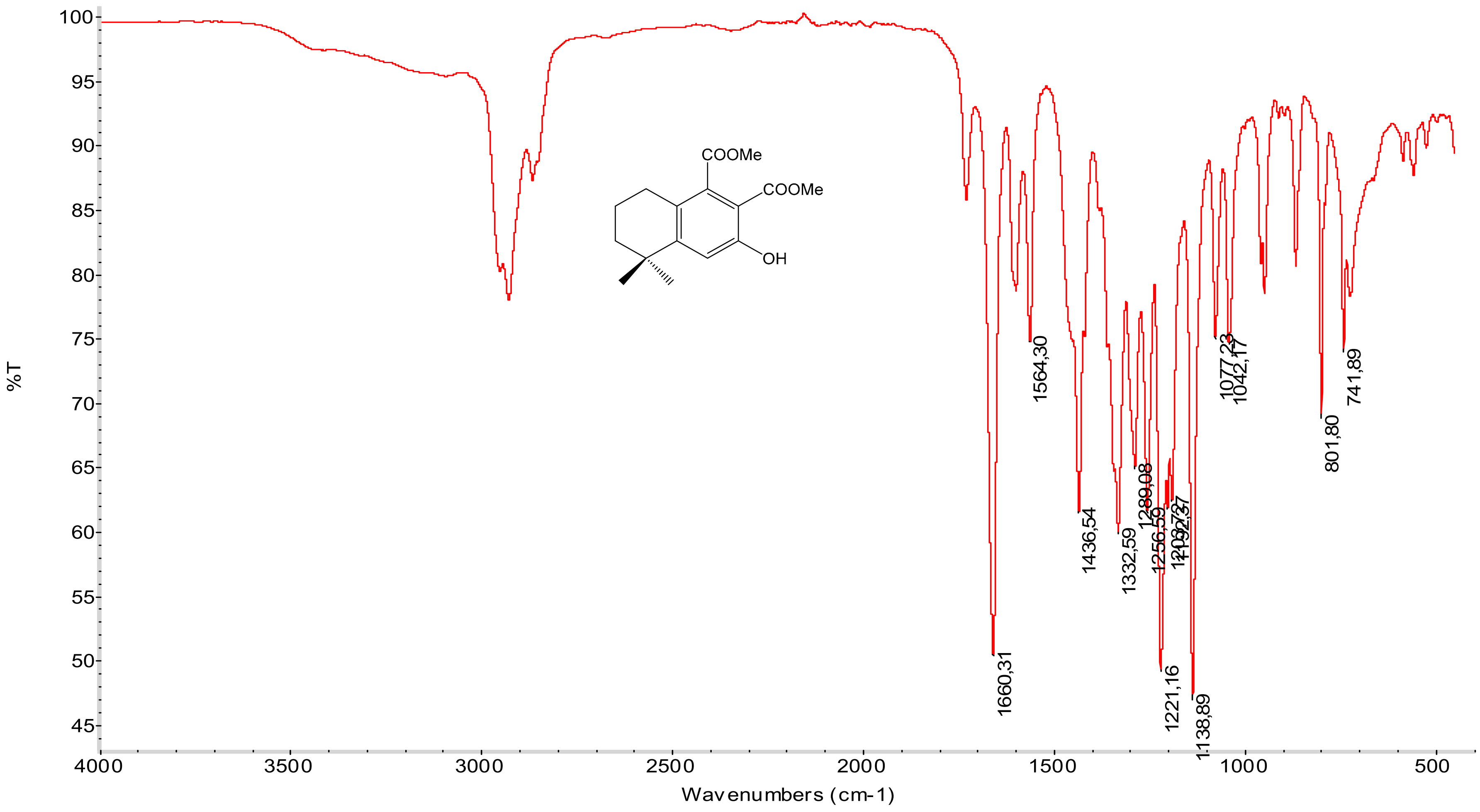

COOMe
COOMe
OH
%T
Wavenumbers (cm-1)
1660,31
1564,30
1436,54
1332,59
1289,08
1256,59
1221,16
1138,89
1077,23
1042,17
801,80
741,89

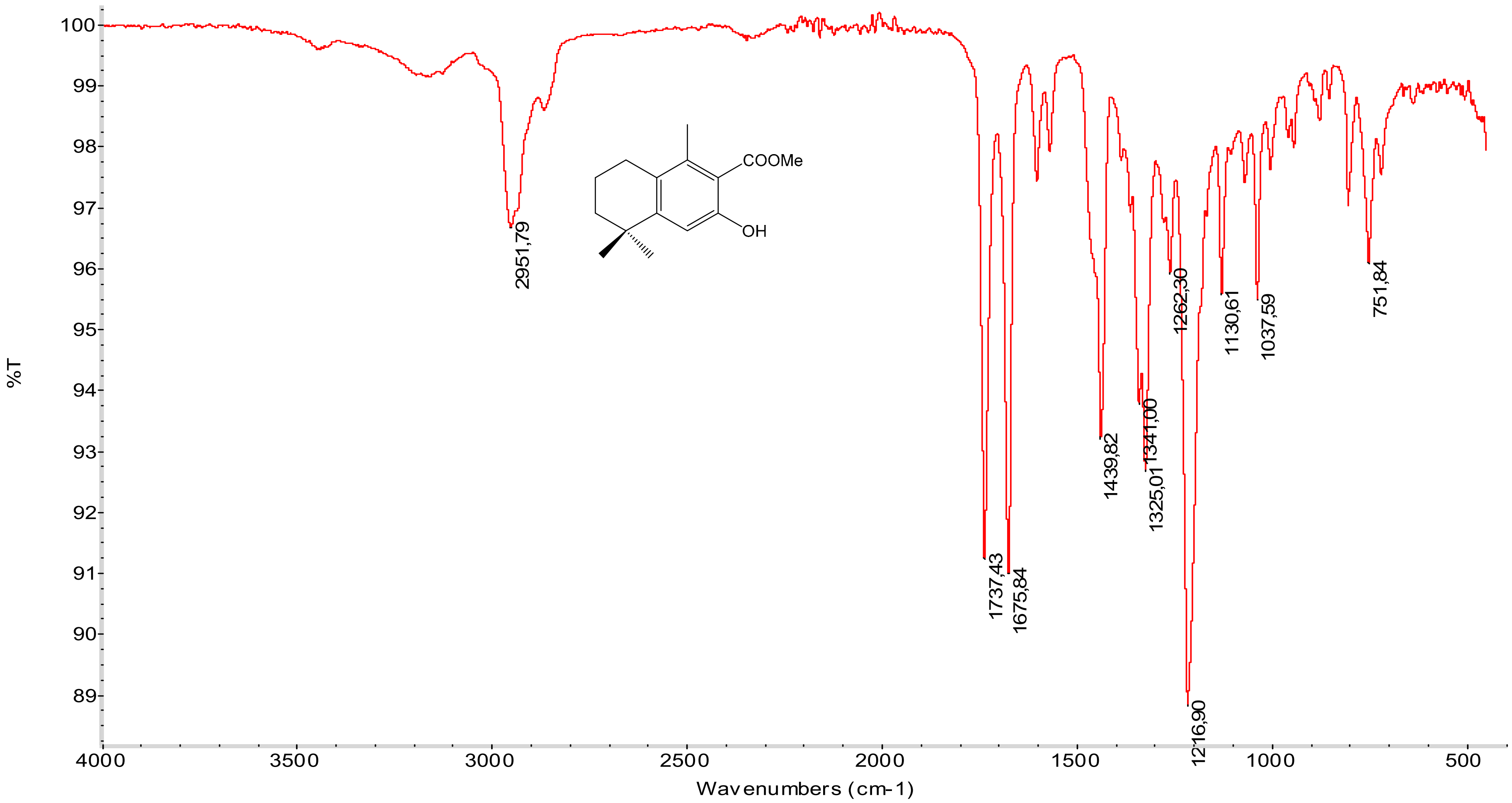
%T
Wavenumbers (cm-1)
COOMe
OH
2951,79
1737,43
1675,84
1439,82
1341,00
1325,01
1262,30
1216,90
1130,61
1037,59
751,84

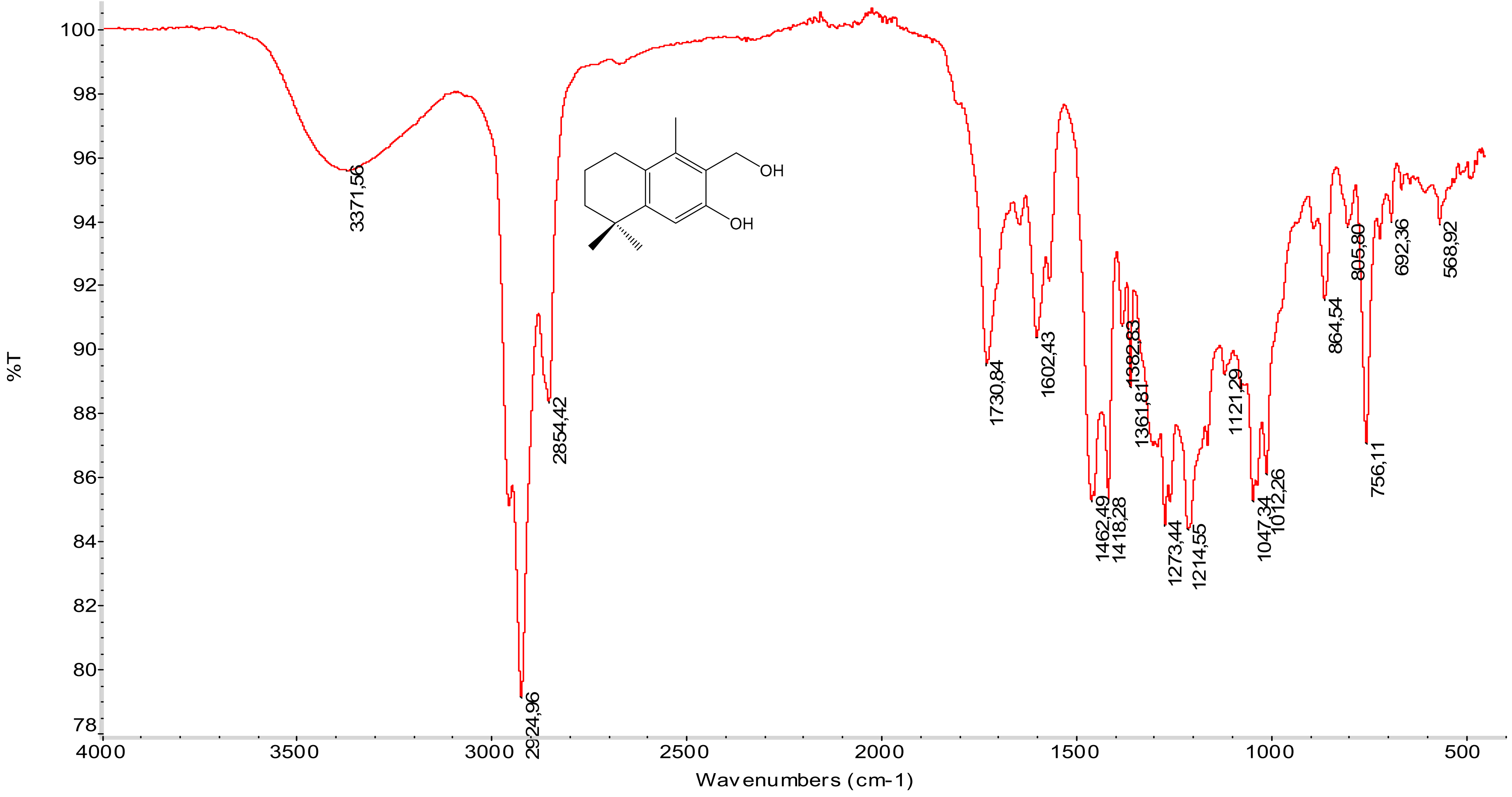
%T
100
98
96
94
92
90
88
86
84
82
80
78
4000
3500
3000
2500
2000
1500
1000
500
Wavenumbers (cm-1)
OH
OH
3371,56
2924,96
2854,42
1730,84
1602,43
1462,49
1418,28
1382,83
1361,81
1273,44
1214,55
1121,29
1047,34
1012,26
864,54
805,80
756,11
692,36
568,92

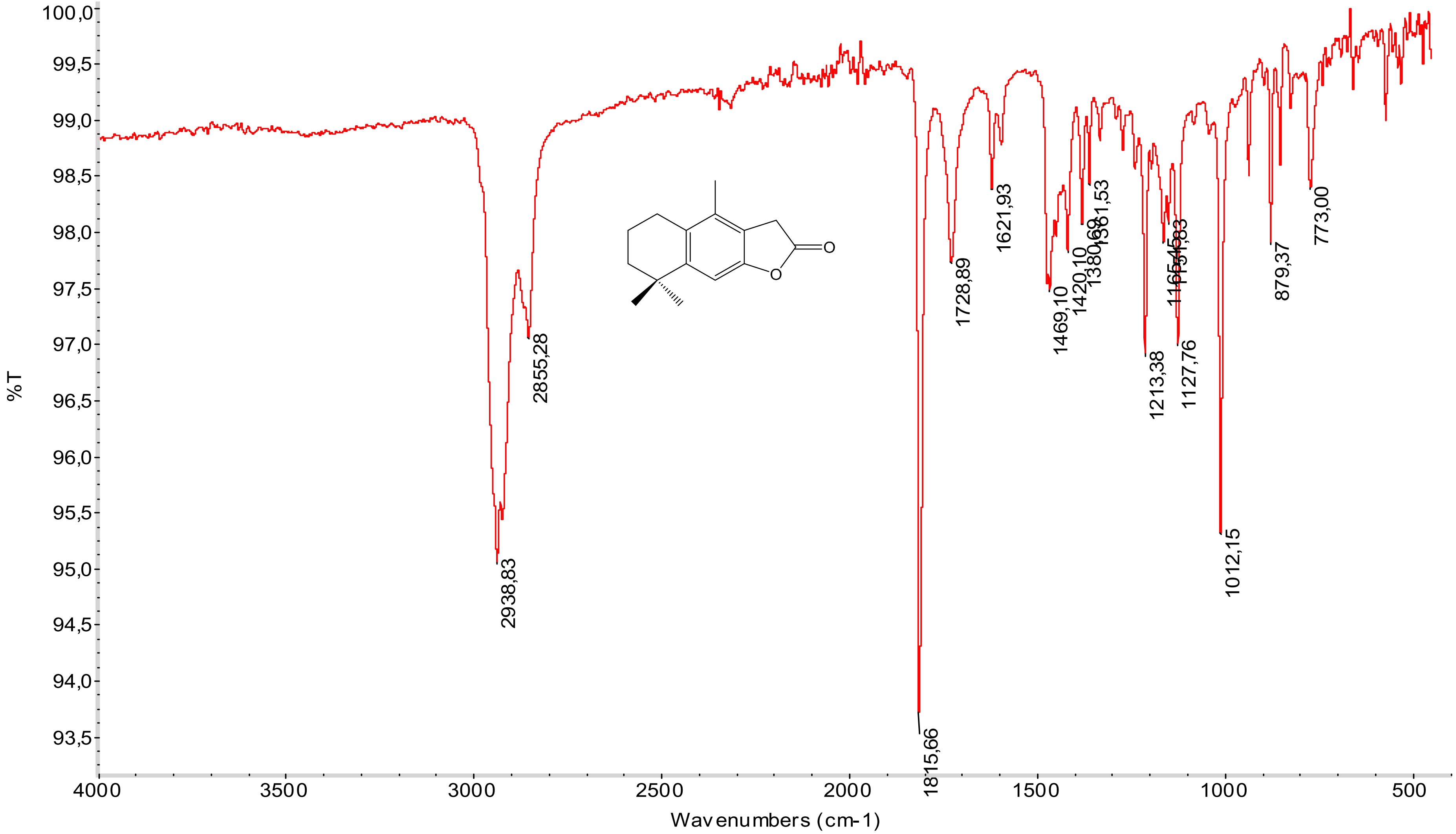

100,0
99,5
99,0
98,5
98,0
97,5
97,0
96,5
96,0
95,5
95,0
94,5
94,0
93,5
%T
4000
3500
3000
2500
2000
1500
1000
500
Wavenumbers (cm-1)
2938,83
2855,28
1815,66
1728,89
1621,93
1469,10
1420,10
1380,69
1361,53
1213,38
1165,45
1151,83
1127,76
1012,15
879,37
773,00
O
O

HRMS spectra of compounds 12,13, 14, 15, and 16

Elemental Composition Report

Page 1

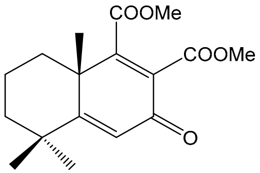

Single Mass Analysis

Tolerance = 25.0 PPM / DBE: min = -20.0, max = 50.0
Element prediction: Off
Number of isotope peaks used for i-FIT = 3

Monoisotopic Mass, Even Electron Ions
202 formula(e) evaluated with 5 results within limits (all results (up to 1000) for each mass)
Elements Used:
C: 0-50 H: 0-100 O: 0-25 23Na: 0-1

20_12_18 1 1284 (5.876)

1: TOF MS ES+
7.08e+006

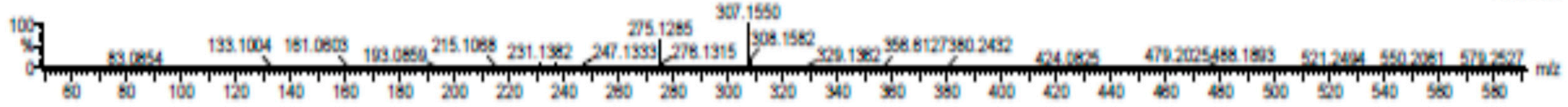

Mass	Calc. Mass	mDa	PPM	DBE	i-FIT	Norm	Conf(%)	Formula
Minimum:				-20.0				
Maximum:		20.0	25.0	50.0				
307.1550	307.1545	0.5	1.6	6.5	748.7	0.019	98.09	C17 H23 O5
	307.1521	2.9	9.4	3.5	752.7	4.020	1.80	C15 H24 O5 23Na
	307.1580	-3.0	-9.8	-5.5	756.3	7.633	0.05	C8 H28 O10 23Na
	307.1604	-5.4	-17.6	-2.5	756.1	7.465	0.06	C10 H27 O10
	307.1487	6.3	20.5	15.5	758.5	9.871	0.01	C24 H19

Elemental Composition Report

Single Mass Analysis

Tolerance = 25.0 PPM / DBE: min = -20.0, max = 50.0
Element prediction: Off
Number of isotope peaks used for i-FIT = 3

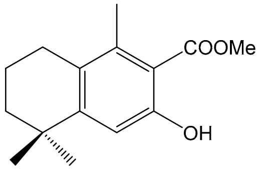

Monoisotopic Mass, Even Electron Ions
146 formula(e) evaluated with 4 results within limits (all results (up to 1000) for each mass)
Elements Used:
C: 0-50 H: 0-100 O: 0-25 23Na: 0-1

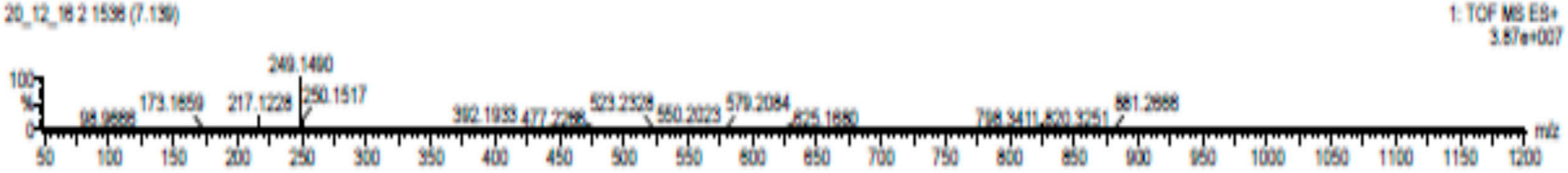

Minimum:				-20.0				
Maximum:		20.0	25.0	50.0				
Mass	Calc. Mass	mDa	PPM	DBE	i-FIT	Norm	Conf(%)	Formula
249.1490	249.1491	-0.1	-0.4	5.5	968.6	0.005	99.48	C15 H21 O3
	249.1467	2.3	9.2	2.5	973.9	5.260	0.52	C13 H22 O3 23Na
	249.1525	-3.5	-14.0	-6.5	982.3	13.653	0.00	C6 H26 O8 23Na
	249.1549	-5.9	-23.7	-3.5	981.1	12.501	0.00	C8 H25 O8

Elemental Composition Report

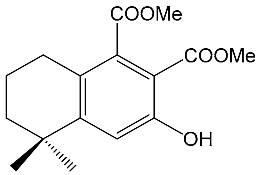

Single Mass Analysis

Tolerance = 25.0 PPM / DBE: min = -20.0, max = 50.0
Element prediction: Off
Number of isotope peaks used for i-FIT = 3

Monoisotopic Mass, Even Electron Ions
211 formula(e) evaluated with 7 results within limits (all results (up to 1000) for each mass)
Elements Used:
C: 0-50 H: 0-100 O: 0-25 Na: 0-1

20_12_16 4 REP 1492 (6.937) 1: TOF MS ES+ 3.30e+007

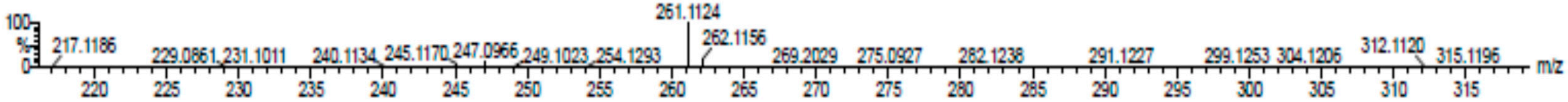

Minimum:				-20.0				
Maximum:		20.0	25.0	50.0				

Mass	Calc. Mass	mDa	PPM	DBE	i-FIT	Norm	Conf(%)	Formula
315.1196	315.1197	-0.1	-0.3	-12.5	685.7	12.835	0.00	H27 O18
	315.1208	-1.2	-3.8	6.5	673.3	0.405	66.69	C16 H20 O5 Na
	315.1174	2.2	7.0	18.5	682.3	9.463	0.01	C25 H15
	315.1232	-3.6	-11.4	9.5	674.0	1.106	33.10	C18 H19 O5
	315.1150	4.6	14.6	15.5	681.5	8.656	0.02	C23 H16 Na
	315.1139	5.7	18.1	-3.5	680.0	7.188	0.08	C7 H23 O13
	315.1267	-7.1	-22.5	-2.5	679.7	6.824	0.11	C9 H24 O10 Na

Elemental Composition Report

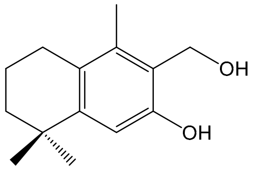

Single Mass Analysis

Tolerance = 25.0 PPM / DBE: min = -20.0, max = 50.0
Element prediction: Off
Number of isotope peaks used for i-FIT = 3

Monoisotopic Mass, Even Electron Ions
128 formula(e) evaluated with 3 results within limits (all results (up to 1000) for each mass)
Elements Used:
C: 0-50 H: 0-100 O: 0-25 Na: 0-1

20_12_16 5 2145 (9.975)

1: TOF MS ES+
6.70e+006

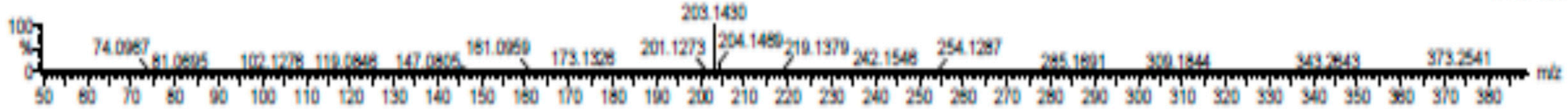

Minimum:				-20.0				
Maximum:		20.0	25.0	50.0				
Mass	Calc. Mass	mDa	PPM	DBE	i-FIT	Norm	Conf(%)	Formula
219.1379	219.1385	-0.6	-2.7	5.5	387.9	0.003	99.68	C14 H19 O2
	219.1361	1.8	8.2	2.5	393.6	5.741	0.32	C12 H20 O2 Na
	219.1420	-4.1	-18.7	-6.5	404.5	16.651	0.00	C5 H24 O7 Na

Elemental Composition Report

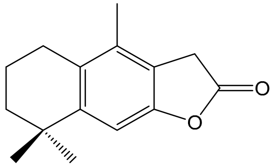

Single Mass Analysis

Tolerance = 25.0 PPM / DBE: min = -20.0, max = 50.0
Element prediction: Off
Number of isotope peaks used for i-FIT = 3

Monoisotopic Mass, Even Electron Ions
138 formula(e) evaluated with 3 results within limits (all results (up to 1000) for each mass)
Elements Used:
C: 0-50 H: 0-100 O: 0-25 Na: 0-1

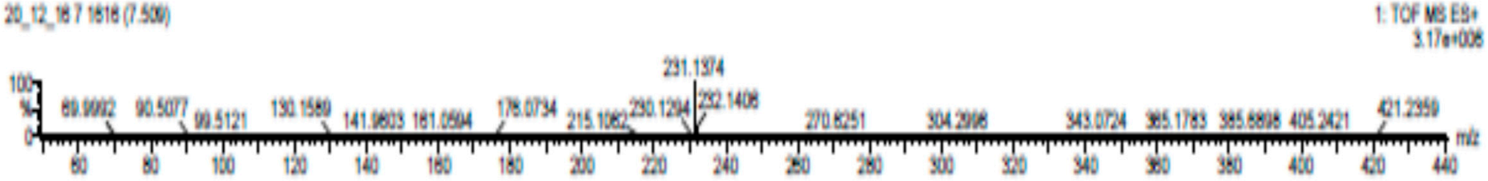

Mass	Calc. Mass	mDa	PPM	DBE	i-FIT	Norm	Conf(%)	Formula
Minimum:				-20.0				
Maximum:		20.0	25.0	50.0				
231.1374	231.1385	-1.1	-4.8	6.5	664.1	0.003	99.70	C15 H19 O2
	231.1361	1.3	5.6	3.5	669.9	5.812	0.30	C13 H20 O2 Na
	231.1420	-4.6	-19.9	-5.5	680.4	16.324	0.00	C6 H24 O7 Na
